# Head of the Firm Gone East!

**Published:** 1873-08

**Authors:** 


### Editorial.

Πρὸς ἠῶ τε καὶ ἡλίου ἀνατολῶς ἐποιέετο τὴν ὁδόν.—Herod. vii. 58.

***Head of the Firm gone East!***

We wonder if old Herodotus ever thought how aptly the above quotation would serve as a sign-board over the doors of most of the firms in this, then *terra incognita*, three thousand years after the hand which wrote it had mouldered into dust. The very aptness of the language goes to show that there is "nothing new under the sun;" that even in the days of Herodotus they "went East!" In compliance with the time-honored custom, and in obedience to the necessities of the case, the JOURNAL hangs out the "notice" over its own door. The July No. went to the devil (printers) before we were able to chronicle our loss, and to explain to our readers the cause of what we fear was but too apparent to their enlightened perceptions, *i. e.*, the absence of its usual amount of *vis vitæ*. Our venerable Senior has departed this life, for a better, we trust, in Europe. His departure at the early dawn was not exactly in the nature of a flight, but rather a masterly retreat, in which much strategic skill was displayed to elude the pursuit of hosts of patients, eager to outflank him.

If our readers will exercise the virtues of forbearance and charity, they will surely be rewarded therefor. They have not been forgotten in this hasty exodus of the Senior Editor, a portion of whose mission to London and Paris, is to secure correspondents for the JOURNAL, that its readers may receive early advices, from first hands, of advances and improvements in medical science and practice. W. H.

We were recently honored with an invitation to attend the Semi-Annual Meeting of the Dubuque County Medical Society, at Dubuque, Iowa. This Society is one of the oldest in the North-west, having attained its full legal majority of twenty-one years; its membership comprises some of the first physicians of the State of Iowa. The proceedings of the meeting were conducted with much dignity and efficiency, by Dr. Jas. C. Lay, Pres., and Dr. Luck, Sec., and comprised very interesting reports of cases, by Drs. Staples, Watson, McCluer, Smith, Missling, Luck and others. The discussion of these gave abundant evidence to show that the best in our profession are not all in the large cities, and that our professional brethren of "the beautiful land" are keeping step to the progress of the times, and in the front rank too. The report of the Board of Censors, by Dr. Asa Horr, Chairman, rejecting two applications for membership, on the ground of professional incompetency and questionable conduct, was dignified in its tone, and temperate in language, but very firm and decided in its expression. We were entirely satisfied that scientific progress and professional ethics are perfectly safe in the hands of our neighbors "on the other side of the river." This Society sustains, and to the best of our knowledge originated, an institution deserving adoption by every other medical society in the land, we might say in the world, *i. e.*, a "Black Book," in which are recorded from time to time, by any member so desiring, the names of all patients willfully and maliciously negligent of their reciprocal obligations to their physicians. This "Black Book" remains in the custody of the Secretary, subject to inspection by the members at their pleasure—who, by thus putting on record the authors of their misfortune, protect each other from the danger of similar disaster.

There are in every community swarms of social sponges, vulgarly termed dead-beats, who, scorning to pick a pocket or rob a bank, have no hesitation in robbing physicians of their time and services.

For the preservation of the names of all such from unmerited oblivion, we commend to all Medical Societies the institution of the "Black Book." W. H.

The attention of our readers is especially directed to the history of a case of rheumatic endocarditis, reported in this No. of the JOURNAL by Dr. H. A. Johnson, Professor of Diseases of the Respiratory and Circulatory Organs in the Chicago Medical College. For the conduct of this case, Dr. Johnson has been most unjustly assailed in the columns of one of the secular papers by a member of the patient's family.

Those who know Dr. Johnson, personally or professionally, will ask of him no explanation or defense—his life and practice preclude the necessity for either. Those who do not know him, will find that the report is its own justification. W. H.

OFFICE OF THE MEDICAL REGISTER AND DIRECTORY OF THE UNITED STATES
No. 115 South Seventh Street,
*Philadelphia*, *June* 6, 1873.

DR. J. ADAMS ALLEN, Chicago, Ill.:

*Dear Sir*—It is *very* important to medical men that they appear in the Medical Register and Directory of the United States, with their proper educational and professional status. Please urge it on your readers, in your next number, to reply, *at once*, to the circulars sent them; or, if they have not received one, to apply to me. Very truly yours, S. W. BUTLER.

We comply with the above request most cheerfully, and as the most effectual mode of doing so, present to our readers the letter of Dr. Butler, entire, which speaks for itself.

